# Modelling *Candida* infection in zebrafish

**Published:** 2013-09

*Candida albicans* is a common component of human gut and oral microbiota that can sometimes cause infections (candidiasis), particularly in immunocompromised individuals. The effects of *Candida* overgrowth can vary from being relatively benign, as in oral or vaginal thrush, to manifesting as potentially life-threatening systemic infections. There are considerable gaps in the current understanding of host-pathogen interactions underlying candidiasis, in part due to the lack of suitable vertebrate models for intravital imaging. To address this, Robert Wheeler and colleagues have established a new zebrafish model for mucosal candidiasis. The group report that this *in vivo* model recapitulates several of the canonical features of mammalian candidiasis and displays gene expression patterns that confirm recent *in vitro* observations. Combined with the potential for noninvasive imaging, these findings indicate that zebrafish will complement existing models and provide new leverage for studying *Candida* infection. **Page 1260**

